# A Rare Case of Small Bowel Extramedullary Plasmacytomas Presenting With Intestinal Obstruction

**DOI:** 10.7759/cureus.15704

**Published:** 2021-06-16

**Authors:** Lynna Alnimer, Ali Zakaria, Bayan Alshare, Yazan Samhouri, Michael Raphael

**Affiliations:** 1 Department of Internal Medicine, Ascension Providence Hospital-Michigan State University/College of Human Medicine, Southfield, USA; 2 Department of Gastroenterology, Ascension Providence Hospital-Michigan State University/College of Human Medicine, Southfield, USA; 3 Department of Oncology, Barbara Ann Karmanos Cancer Institute, Detroit, USA; 4 Department of Hematology and Oncology, Allegheny Health Network, Pittsburgh, USA

**Keywords:** gastrointestinal obstruction, duodenum, small bowel, extramedullary plasmacytoma, plasmacytoma, plasma cell dyscrasias

## Abstract

Extramedullary plasmacytoma (EMP) is a plasma cell disorder involving soft tissues in the absence of clonal bone marrow involvement or destructive bone lesions. When present in the gastrointestinal (GI) tract, and specifically the small intestine, it can cause a wide range of symptoms including GI bleeding, obstruction, and abdominal pain. The diagnosis is challenging, as it can hold an indolent course, and is infrequently encountered in clinical practice. Diagnosis requires biopsy of the involved organ, which can be obtained during surgery or endoscopy, and other workup to rule out systemic disease and bone marrow involvement. Treatment depends on the primary site of disease involvement and the presence of other features of systemic disease. We report a case of multiple small bowel plasmacytomas in a 51-year-old female who presented with small bowel obstruction. She eventually underwent surgical resection and is currently on chemotherapy awaiting stem cell transplant.

## Introduction

The incidence of extramedullary plasmacytoma (EMP) accounts for 3% of all plasma cell neoplasms (PCNs) and rarely involves the gastrointestinal (GI) tract, accounting for 4-5% of all EMPs [1-3]. GI EMPs are most frequently located in the stomach followed by the liver and colon, whereas the small bowel (duodenum, jejunum, and ileum) is considered a rare location [4]. The median age at diagnosis is 55 to 60 years, with male predominance [2]. A comprehensive review of the literature reported 61 cases of small bowel plasmacytomas (20 in the duodenum, 24 in the jejunum, and 17 in the ileum) [4]. We report a case of a 51-year-old female with a previous history of solitary osseous plasmacytoma of the left ileum who presented with nausea, vomiting, and abdominal pain and was found to have multiple small bowel plasmacytomas.

## Case presentation

**Figure 1:**
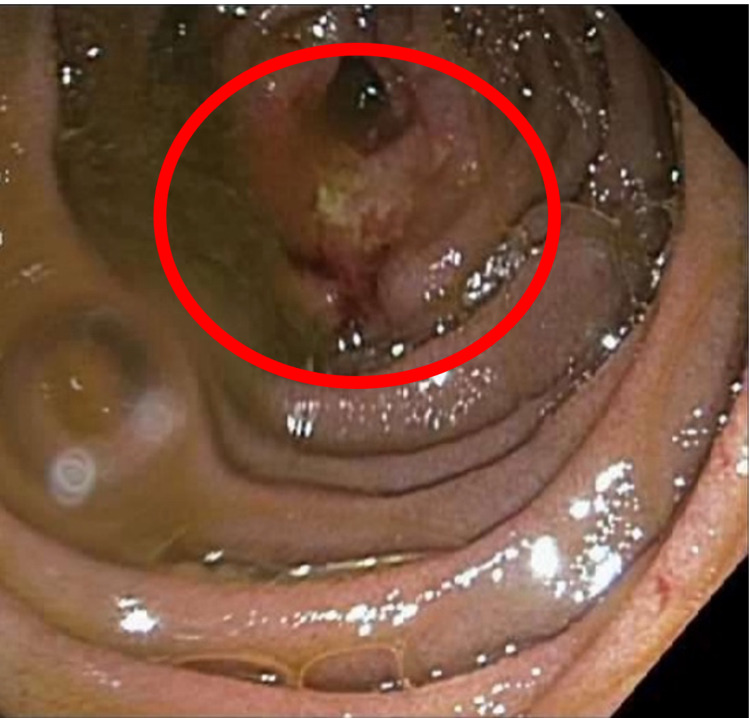
Fungating and ulcerated proximal jejunal lesion identified during push enteroscopy (marked with a red circle).

**Figure 2:**
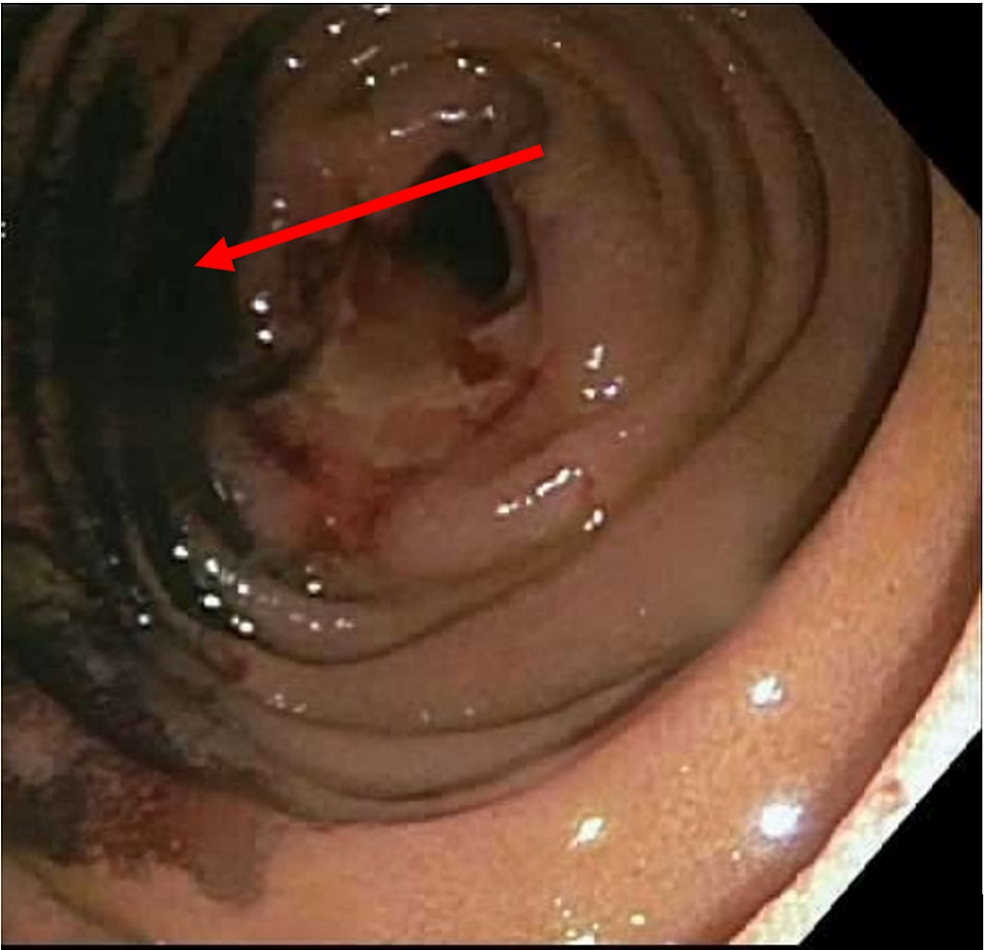
Injection of Spot® Ex (ink tattoo) around the proximal jejunal lesion during push enteroscopy (marked with a red arrow).

A 51-year-old Caucasian female with a medical history of solitary plasmacytoma of the left acetabulum treated with radiation therapy three years ago presented to our gastroenterology outpatient clinic complaining of multiple GI symptoms including abdominal pain, nausea, vomiting, early satiety, and bloating of two months’ duration. Symptoms were worse with eating. She had an unintentional weight loss of 30 lbs. Physical examination of the abdomen was unremarkable. A computed tomography (CT) angiogram of the abdomen and pelvis revealed multiple soft tissue masses identified within the small bowel causing partial small bowel obstruction. Esophagogastroduodenoscopy (EGD) was performed, which was unremarkable. A small bowel push enteroscopy revealed a large fungating and ulcerated mass in the proximal jejunum (Figure 1), which was biopsied using cold forceps and tattooed using 3 mL of Spot® Ex (Figure 2). Initial laboratory tests showed a WBC count of 5.11 K/mcL, absolute neutrophil count of 7.86 K/mcL, hemoglobin of 15.0 gm/dL, and platelets of 498 K/mcL.

**Figure 3:**
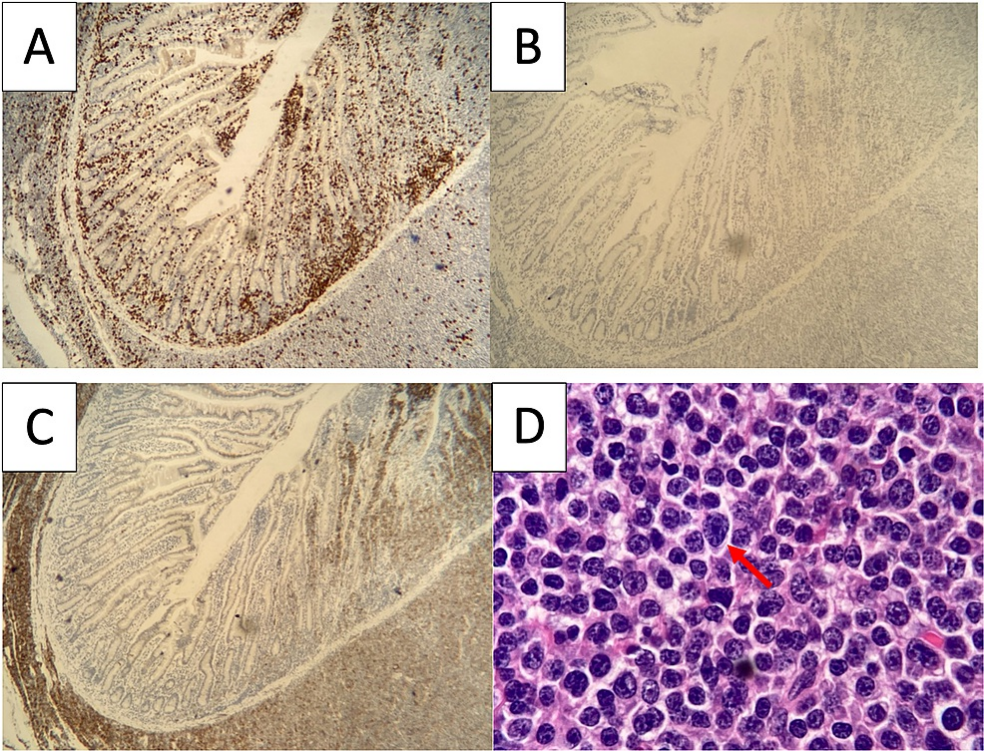
Histopathology findings of the proximal jejunal lesion. (A) CD3 positive (x80). (B) CD 20 positive (x80). (C) CD 138 positive (x80). (D) Small bowel specimen showing plasma cells (marked by red arrow) (x800).

She underwent a diagnostic open laparotomy with resection of two separate masses in the proximal small bowel (measuring 7.5 and 4.0 cm) and one mass (5.5 cm) in the distal small bowel. Immunohistochemistry analysis of the tissue sample was positive for CD138, CD79a, and MUM1, and negative for CD20 consistent with PCN (Figures 3A-3D). Flow cytometry showed a large population of cells expressing CD38 and CD56. In situ staining for kappa and lambda showed lambda light chain restriction in the malignant plasma cells. Fluorescent in situ hybridization (FISH) revealed 17p deletion and monosomy 13. Four out of 13 lymph nodes were involved by the neoplasm.

After the identification of a plasma cell disorder, further workup was performed. Serum immunofixation showed a faint immunoglobulin (Ig) A lambda restriction. Serum protein electrophoresis (SPEP) showed a faint IgA lambda band. Free light chains showed slightly elevated lambda light chain at 3.73 mg/dL, and quantitative immunoglobulins were normal (IgG: 719; IgA: 141; and IgM: 71 mg/dL). Fluorodeoxyglucose positron emission tomography/CT (FDG-PET/CT) scan revealed two enlarged lymph nodes in the left mesentery measuring 1.4 cm each, with a maximum standardized uptake value (SUV) of 3.9. There was increased metabolic activity around the two enteric anastomoses. There was no evidence of destructive bone lesions. Findings included the presence of a sclerotic focus involving the lateral right sixth rib, an increased metabolic activity involving the superior right acetabulum, and a stable previously irradiated lesion in the left hemipelvis with lytic and sclerotic appearance without FDG activity. She underwent a bone marrow aspirate and biopsy with an adequate sample. The biopsy showed normo-cellular bone marrow for age with no clusters of plasma cells or lymphoid aggregates, and the aspirate showed less than 1% of plasma cells with polytypic light chain expression and no evidence of clonality. Cytogenetics showed a normal female karyotype.

Our patient developed small bowel EMPs years after the diagnosis of solitary bone plasmacytoma with no evidence of clinical or biologic features of multiple myeloma. In addition to the surgical management, she was started on induction systemic therapy with four agents, daratumumab, lenalidomide, bortezomib, and dexamethasone, with the plan for consolidation with autologous stem cell transplant followed by maintenance therapy.

## Discussion

According to the International Myeloma Working Group (IMWG), extramedullary solitary plasmacytoma is defined as a biopsy-proven solitary lesion of bone or any other soft tissue with evidence of clonal plasma cells [5]. The criteria for diagnosis involve normal bone marrow with no evidence of clonal plasma cells, absence of CRAB (hypercalcemia, renal insufficiency, anemia, and lytic bone lesions) criteria, and normal skeletal survey and MRI of the spine and pelvis [5]. Extramedullary solitary plasmacytomas can occur as a primary disease or secondary to multiple myeloma. In addition, patients with the primary disease can progress to develop multiple myeloma. It is not clear why some patients progress while others do not. One theory attributes this phenomenon to differences in chemokine receptors and cellular adhesion molecules expressed on malignant plasma cells [6].

Although secondary EMPs are generally more common, small bowel plasmacytoma cases described in the literature are mostly primary [4]. This could be related to the indolent course associated with small intestine EMPs, yet the clinical presentation widely varies based on site, size, and involvement of surrounding structures [3,4]. Patients can present with symptoms related to mass effect on the surrounding organs including abdominal pain, which is by far the most common symptom reported, intestinal obstruction, or painless jaundice [3,4,7]. Similar to our patient’s presentation, symptoms such as nausea, vomiting, weight loss, and early satiety can occur with intraluminal disease, which can mimic other GI malignancies such as adenocarcinoma or lymphoma [3]. Small bowel involvement can lead to signs of intussusception, while gastric involvement may lead to bleeding and melena [8]. Malabsorption, diarrhea, and rarely ileocolic fistula can occur when the disease involves the ileum [9,10].

The workup includes routine laboratory tests such as complete blood cell count (CBC) and a complete metabolic panel (CMP), in addition to a biopsy-proven extramedullary tumor showing clonal plasma cells, advanced imaging showing no lytic lesions, and a bone marrow biopsy free of clonal plasma cells. Some patients with EMPs might have a small amount of monoclonal protein, usually IgA, in either the serum or urine, in the absence of bone marrow disease [5]. This usually resolves with treatment. Plasma cells are positive for CD138 and CD38 and show light chain restriction. In our patient’s case, she developed small bowel EMPs years after the diagnosis of solitary bone plasmacytoma with no evidence of clinical or biologic features of multiple myeloma, hence the systemic therapy and stem cell transplant evaluation.

Methods to obtain tissue diagnosis depend on the anatomic location. For instance, endoscopic ultrasound-fine needle aspiration (EUS-FNA) has been used in the diagnosis of EMP in the pancreas, liver, and gallbladder [11,12]. Surgical resection or open/laparoscopic biopsy can be used to confirm the diagnosis. Using small bowel enteroscopy, we were able to recognize the presence of a small bowel mass, but the diagnosis was confirmed with surgical resection. This reflects the diagnostic yield of push enteroscopy yet addresses its limitations when it comes to the presence of complicated multiple masses. Approximately 30% of patients may progress to PCN [13]. Progression to PCN is extremely rare in small intestine EMP, with only three cases reported in a review by Lopes da Silva [4,14,15].

There are no clear guidelines on the management of intra-abdominal EMP as most data described in the literature are from case reports and small-volume case series [3]. Treatment strategies are usually discussed among a multidisciplinary team consisting of hematology/oncology, surgery, and radiation oncology. The treatment of choice for solitary plasmacytoma is radiotherapy, 40-50 Gy over a four-week period, with the intention to cure [16]. Another valid option for small bowel plasmacytoma is complete surgical resection of the mass since diagnosis tends to require surgery anyway and it carries the lowest recurrence rate [4]. Systemic chemotherapy is similar to that used in multiple myeloma and it is more commonly used when EMP presents secondary to multiple myeloma. In small bowel plasmacytoma, whether it is a primary disease or extramedullary spread, the treatment starts with addressing the local complications caused by the mass itself.

## Conclusions

Small bowel plasmacytoma is a rare entity that can present with debilitating symptoms. Although rare, gastroenterologists should keep a high index of suspicion for intra-abdominal EMP as a differential diagnosis in the setting of nonspecific GI symptoms with a known history of solitary plasmacytoma of the bone or in the setting of multiple myeloma. Definitive diagnosis is made after tissue sampling either endoscopically or surgically. However, this case highlights the diagnostic value of push enteroscopy as gastroenterologists are able to identify the lesions and mark the borders with tattooing, which is a relatively safe procedure.
